# Recognition of Foot-Ankle Movement Patterns in Long-Distance Runners With Different Experience Levels Using Support Vector Machines

**DOI:** 10.3389/fbioe.2020.00576

**Published:** 2020-06-11

**Authors:** Eneida Yuri Suda, Ricky Watari, Alessandra Bento Matias, Isabel C. N. Sacco

**Affiliations:** Physical Therapy, Speech and Occupational Therapy Department, School of Medicine, University of São Paulo, São Paulo, Brazil

**Keywords:** running, machine learning, running experience, biomechanics, fuzzy logic

## Abstract

Running practice could generate musculoskeletal adaptations that modify the body mechanics and generate different biomechanical patterns for individuals with distinct levels of experience. Therefore, the aim of this study was to investigate whether foot-ankle kinetic and kinematic patterns can be used to discriminate different levels of experience in running practice of recreational runners using a machine learning approach. Seventy-eight long-distance runners (40.7 ± 7.0 years) were classified into less experienced (*n* = 24), moderately experienced (*n* = 23), or experienced (*n* = 31) runners using a fuzzy classification system, based on training frequency, volume, competitions and practice time. Three-dimensional kinematics of the foot-ankle and ground reaction forces (GRF) were acquired while the subjects ran on an instrumented treadmill at a self-selected speed (9.5–10.5 km/h). The foot-ankle kinematic and kinetic time series underwent a principal component analysis for data reduction, and combined with the discrete GRF variables to serve as inputs in a support vector machine (SVM), to determine if the groups could be distinguished between them in a one-vs.-all approach. The SVM models successfully classified all experience groups with significant crossvalidated accuracy rates and strong to very strong Matthew’s correlation coefficients, based on features from the input data. Overall, foot mechanics was different according to running experience level. The main distinguishing kinematic factors for the less experienced group were a greater dorsiflexion of the first metatarsophalangeal joint and a larger plantarflexion angles between the calcaneus and metatarsals, whereas the experienced runners displayed the opposite pattern for the same joints. As for the moderately experienced runners, although they were successfully classified, they did not present a visually identifiable running pattern, and seem to be an intermediate group between the less and more experienced runners. The results of this study have the potential to assist the development of training programs targeting improvement in performance and rehabilitation protocols for preventing injuries.

## Introduction

Running is a very popular activity and its practice has been increasing in the last decades because of its accessibility and several benefits (Lee et al., 2017). It is a repetitive activity which results in minor load variations at each step (Davis and Futrell, 2016). These repeated loads that occur during running have beneficial effects over the foot musculoskeletal structures, such as increases in foot muscle volume and cross-sectional area, and in bone density (Garofolini and Taylor, 2019). However, repetitive loading can also make musculoskeletal tissues from the lower limbs more susceptible to cumulative overload and, therefore, overuse injuries (Davis and Futrell, 2016).

Notably, the foot-ankle forms a dynamic link between the body and the ground (Rodgers, 1988), being the first segment to provide this interaction and being responsible for the accommodation for the irregularities of the supporting surfaces. Additionally, the foot-ankle complex contributes to the dissipation of energy returned from the ground and to the attenuation of high impact forces during foot strike in running by having many kinematic adjustments in its more than 33 joints, participating in propulsion generation during push-off (Holowka and Lieberman, 2018), and storing and releasing elastic energy during stance phase (Holowka and Lieberman, 2018; Kelly et al., 2018). Altogether, these features make the structure and function of the foot-ankle complex extremely important for running practice. There are evidences that barefoot running, which enhances strength of the plantarflexors and foot intrinsic muscles (Lieberman, 2012), might serve as protection against knee injuries (Altman and Davis, 2016), one of the most common running-related injury (van Gent et al., 2007). Therefore, the study of possible changes that might occur in foot-ankle biomechanics with running practice gains importance.

Running experience appears to be protective against injuries (Macera, 1992; Nielsen et al., 2012; van der Worp et al., 2015; Hulme et al., 2017; Linton and Valentin, 2018). Videbæk et al. (2015) showed that novice runners have a significantly greater risk of injury than recreational runners, who run regularly and participate in short races (less than 10 km), showing an incidence of 17.8 per 1000 h of running against 7.7, respectively. Besides that, novice runners have the majority of injuries in the lower leg (34.7%), 3.5% in the foot and 8.2% in the ankle, while recreational runners present the majority of injuries in the knee (26.3%), 10.1% in the foot and 7.8% in the ankle (Kluitenberg et al., 2015). Those differences in injury incidence and distribution point out that these might be distinct populations, who could present different running mechanics that can be related to experience level and could be a protective factor for running-related injuries.

However, most studies classify experience based solely on years of practice, or just classify the subjects between “novice” or “experienced,” leaving the reasons behind this possible protective effect unknown. Although development of expertise can be achieved through deliberate practice, i.e., by performing a task in a way that provides effective skill acquisition (Ericsson et al., 1993), defining running experience just as years of practice does not take into account the structure of the deliberate practice (Iglesias et al., 2010). In this context, Roveri et al. (2017) developed a decision support system to classify experience in the deliberate practice of running that takes into account the structure of the practice, including training frequency, training volume, years of practice, and participation in races as inputs in the algorithm. Therefore, using this classification approach could provide a more complete and objective measure of experience levels, allowing the study of how the biomechanical adaptations in running might be transitioning across the experience acquisition process. Furthermore, future studies could take advantage of these findings to investigate what are the implications of the potential differences in biomechanics to running performance and injuries.

Some studies suggest that running experience does not influence running biomechanics. Agresta et al. (2018) did not find any influence of years of running experience in trunk and lower limb kinematics, spatiotemporal variables, nor GRF variables during running. Similarly, Schmitz et al. (2014) did not find differences between novice and experienced runners in impact peak, loading rate, nor peak hip adduction angle during running. However, Clermont et al. (2017) compared 3D running kinematics of the pelvis, hip, knee and ankle of recreational and competitive runners by means of a combination of principal component analysis and a SVM classifier and determined that it is possible to distinguish both groups based on the differences in pelvic tilt, knee flexion and ankle eversion. Hence, there is evidence that multivariate analysis combined with machine learning approaches can be an effective analysis method for identifying mechanical patterns of running. Nevertheless, there is still a lack of understanding of how the different segments of the foot-ankle complex participate in these biomechanical adaptations, given that there is a functional importance of this multi-joint complex during running regarding energy dissipation, attenuation of forces, energy storage and release during stance, and propulsion generation.

In this context, studying the effects of experience on the mechanical behavior of the foot-ankle can contribute to the better understanding of how it is influenced by the skill acquisition. This understanding could give insights on what type of training or rehabilitation protocol could enhance performance or prevent and treat injuries. Therefore, the main purpose of this study is to determine if it is possible to separate and classify groups with distinct levels of experience, determined by a system that takes into account different aspects of running practice, based on foot-ankle kinematics and kinetic patterns, and impact variables. We hypothesize that there will be different foot-ankle mechanical patterns that can distinguish between levels of running experience, identified by a machine learning approach.

## Materials and Methods

This study was a retrospective secondary analysis of a subset of data from a larger randomized clinical trial approved by the Research and Ethics Committee of the School of Medicine, University of São Paulo (Protocol no. 031/15) and registered at ClinicalTrials.gov (Identifier NCT02306148).

### Participants

Data from 78 recreational long-distance runners were selected based on the availability for this analysis, since this study is a secondary analysis from a larger randomized clinical trial. All runners consented to participate after receiving information on all details of the study. Participants were between 18 and 55 years of age, ran between 20 and 100 km per week, and had no lower limb injury or pain in the 3 months prior to assessment. Participants were excluded if they were under any physical therapy treatment at baseline, had a history of using minimalist shoes or barefoot running, presented any orthopedic or neurologic impairment or major vascular complication, had previous lower-limb surgery, or had diabetes mellitus.

Running experience was classified by a fuzzy decision-support system developed by Roveri et al. (2017), that is composed by two Mamdani subsystems based on expert running coaches’ knowledge. The first subsystem uses the training frequency and volume as inputs, which are transformed into linguistic variables: (i) too low, (ii) low, (iii) medium, or (iv) high for each one of the inputs. These linguistic variables are combined to generate a score (0–10) of quality of practice. In the second subsystem, the quality of practice serves as input, along with the number of competitions and practice time, also transformed in linguistic variables: (i) very bad, (ii) bad, (iii) medium, (iv) good, or (v) very good for quality of practice; (i) few, (ii) medium or (iii) many for number of competitions; (i) very short, (ii) short, (iii) moderate, or (iv) long for practice time. The second subsystem generates, then, the final score of running experience. The running experience score (*x*) for was used to classify the subjects as less experienced (*x* < 5.0), moderately experienced (5.0 ≤ *x* ≤ 7.0), or experienced (*x* > 7.0). The characteristics of the subjects according to the experience level and anthropometry are shown in Table 1.

**TABLE 1:** Mean and standard deviation of participants’ characteristics from the studied groups.

	Less Experienced (*n* = 24)	Moderately experienced (*n* = 23)	Experienced (*n* = 31)	*P*
Age (years)	40.1 ± 5.3	40.6 ± 7.1	41.8 ± 7.0	0.614^†^
Height (m)	1.66 ± 0.09	1.71 ± 0.09	1.69 ± 0.09	0.097^†^
Body mass (kg)	71.1 ± 15.4	74.7 ± 10.4	67.1 ± 11.6	0.136^†^
Body mass index (kg/cm^2^)	25.6 ± 3.5^a^	25.2 ± 3.1	23.3 ± 2.5^a^	0.017^†^*
Sex (% women)	41.7	60.9	48.4	0.409^‡^
Training frequency (times/week)	3.1 ± 0.6^bc^	3.9 ± 1.0^b^	4.2 ± 1.6^c^	<0.001^†*¥^
Training volume (km/week)	20.0 ± 5.6^d^	29.1 ± 10.3^e^	54.1 ± 38.0^de^	<0.001^†*¥^
Quality of practice (fuzzy system score)	3.4 ± 0.9	4.6 ± 1.2	6.7 ± 1.6	<0.001^†*¥^
Years of practice (years)	2.8 ± 2.9^f^	7.7 ± 11.0	9.0 ± 5.8^f^	0.006^†^*
Participation in races	18.8 ± 26.0^g^	31.1 ± 29.4	53.1 ± 56.9^g^	0.012^†^*
Running experience level (fuzzy system score)	3.2 ± 0.8	5.9 ± 0.7	7.9 ± 0.6	<0.001^†*¥^
Running velocity at data collection (km/h)	9.4 ± 1.4	9.8 ± 1.5	9.7 ± 1.0	0.639^†^

^†^ANOVA followed by Bonferroni *post hoc* tests. ^‡^Chi-square test. *Statistically significant differences. ^¥^Statistically significant differences between all studied groups. ^*a,b,c,d,e,f,g*^Statistically significant differences between the groups.

### Data Collection

Foot biomechanics were assessed during barefoot running at a self-selected speed on an instrumented treadmill, which was leveled with the ground and embedded with two force plates in tandem position (AMTI Force-Sensing treadmill AMTI, Watertown, EUA; force plates at 1000 Hz). Foot kinematics were acquired using eight infrared cameras (Vicon^®^ VERO, Vicon Motion System, Ltd., Oxford Metrics, United Kingdom; at 200 Hz) and 16 retroreflective markers (10 mm in diameter) were placed on the dominant foot according to the Rizzoli Orthopedics Institute Foot Model (Leardini et al., 2007; Portinaro et al., 2014). Subjects underwent a warmup period for habituation to the treadmill and laboratory environment, after which, kinematic data was recorded for 30 s in order to acquire at least 10 step cycles of the assessed limb. There was no statistically significant difference for running velocity across groups (Table 1).

### Data Processing

The origin of the laboratory coordinate system was defined as one corner of the force plate and all segments were modeled as rigid bodies with the local coordinate system coinciding with the anatomical axes. All joints were considered to have a spherical shape (three rotational degrees of freedom), with rotations of each segment reported relative to the neutral positions defined during the initial static standing trial. All joint rotations were calculated based on the International Society of Biomechanics recommendations (Wu et al., 2002).

Kinematic and GRF data were analyzed and processed using a zero-lag, fourth-order Butterworth low-pass filter with cutoff frequencies of 15 and 100 Hz, respectively, based on residual analysis (Winter and Patla, 1997).

We extracted the eight kinematic time series from the following joints: ankle in all three movement planes; medial longitudinal arch in sagittal plane (Caravaggi et al., 2019); 1^st^ metatarsophalangeal joint in sagittal plane (Met-Hal); the angle between the calcaneus and metatarsal bones (Cal-Met) in all planes.

Calculation of joint kinematics and kinetics were performed using Visual3D software (C-motion, Kingston, ON, Canada). A bottom–up inverse dynamics method was used to calculate the net ankle moment and power in the sagittal plane, with the human body modeled as 2 linked segments (foot and shank) and the inertial properties were based on Dempster’s standard regression equations. Net ankle moment and power were calculated for the stance phase.

**FIGURE 1:**
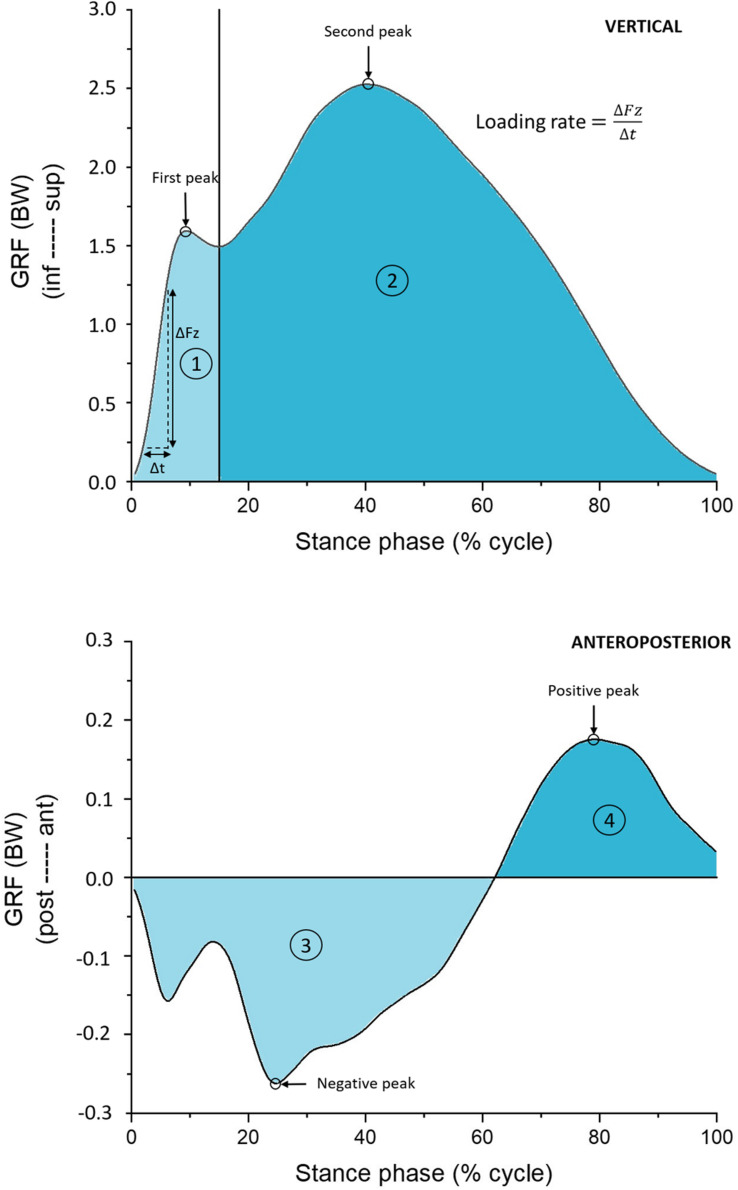
Vertical and anteroposterior (AP) ground reaction forces (GRF) showing the extracted variables: force peaks for both vertical and AP forces, and loading rate for the vertical component. Loading rate was determined as the slope of the line between 20 and 80% of the first vertical peak. The colored areas correspond to the calculated impulses: 1 – from the beginning of the stance phase to the valley after the first vertical peak (light blue area, upper graph); 2 – from the valley after the first vertical peak to the end of the stance phase (blue area, upper graph); 3 – decelerating phase (light blue area, bottom graph); 4 – accelerating phase (blue area, bottom graph).

All nine analyzed GRF variables were normalized by each runner’s body weight. From the vertical component, it was extracted the first and second peaks, calculated the loading rate (the force rate between 20 and 80% of the magnitude between the foot contact and the first peak), and the impulses from the beginning of the stance phase to the second peak, and from the second peak to the end of stance phase (Figure 1). From the anteroposterior component, it was extracted the negative and positive peaks and calculated the impulses from the decelerating and accelerating phases (Figure 1).

### Machine Learning Analysis

#### Feature Extraction

The stance phase of all kinematic (eight variables) and kinetic (ankle sagittal moment and power) time series were determined by means of the vertical GRF using a 10 N threshold and normalized in time to 101 points. Then, the data waveforms (10 waveforms) were averaged across 10 consecutive stance phases of the dominant limb (101 data points per axis direction for each joint kinematics and kinetic waveform), combined into a 78 × 1010 matrix (78 runners X 1010 waveform data point), and standardized to a mean of 0 and a standard deviation of 1 (Kettaneh et al., 2005). Given the large number of dependent variables and potential for redundancy of data, this data matrix underwent a principal component analysis (PCA), resulting in 77 principal components (PCs). PCA is an orthogonal transformation technique used to convert a set of variables into a set of linearly uncorrelated variables by determining new bases (PCs) that maximize the variability in the original data set (Abdi and Williams, 2010). These PC scores were combined with nine standardized discrete GRF variables, resulting in a total set of 86 potential predictor variables.

#### Classification Procedures

The potential predictors were used as inputs for creating SVM models to classify the runners into each experience level group, using a one-vs.-all approach. The SVM approach was chosen because of its capability of overcoming the problem of high dimensionality with high discriminative power for group classification, even in cases with small sample sizes (Verplancke et al., 2008; Son et al., 2010). The SVM algorithm (Noble, 2006) defines an optimal separating hyperplane, creating a maximum-margin of separation between binary classes in a dataset. For that, the SVM projects the input feature’s data into a higher dimensional space using kernel functions, and then, based on the data points located closest to the separating hyperplane (support vectors), constructs a linear hyperplane in this transformed space, which can be projected back to the original data space. In order to deal with possible misclassifications (datapoints in the wrong side of the separating hyperplane), SVM uses the soft margin concept, which allows these errors without affecting the final result. The trade-off between margin width and misclassification rate is defined by the C-parameter, wherein different values for C (0.1, 1, 10, 100, 1000) were used in the evaluation to test the dependence of the approach on the C-parameter. A linear kernel function was applied to the SVM algorithm, since it is less prone to overfitting to the dataset, and the current study has a limited sample size.

A sequential forward selection algorithm was applied for the identification of relevant features, in which a subset of potential predictor variables was defined by sequentially adding one new feature at a time to the SVM model, and the feature subset that rendered the best classification performance was selected. In order to assess the generalization performance of the classifier in identifying the label of unknown data and to avoid data overfitting, a 10-fold cross-validation was performed (Fukuchi et al., 2011), wherein the runners were randomly divided into 10 subsets, stratified proportionally by the experience level class. The SVM algorithm was trained by removing one subset at a time and the resulting model was applied to the holdout subset to determine the cross-validation performance. The evaluation function selected the feature that provided the highest Matthew’s correlation coefficient (MCC) as the first criteria, because the group distribution becomes unequal in a one-vs.-all approach (Chicco and Jurman, 2020). When it was not possible to calculate the MCC, because of absence of predicted cases in one class, the feature with highest Cohen’s *d* effect size and highest performance accuracy was selected. This process was repeated for all input variables in a greedy search approach, applying each C-parameter value, and the model with the highest MCC was chosen as the final classification model.

#### Classifier Performance and Interpretation

In order to assess the performance of the SVM models, the cross-validated classification results were used to calculate the MCC, accuracy, precision, recall, and F1-score. A critical binomial test indicated the minimum significant accuracy level for each model, considering a distribution probability equal to the ratio of cases in each experience level class and a confidence level of 0.95.

The squared coefficients of correlation between the PC scores and the joint kinematic and kinetic waveforms was calculated as a measure of proportion of variance of the data that was explained by the selected PCs (Abdi and Williams, 2010), and the relative proportion in each joint and axis was used to help with the interpretation of the selected PC features. The data waveforms were reconstructed based on the selected PCs for each model and the Cohen’s *d* effect size between each group and the remaining runners was calculated. The regions of the waveforms with at least a medium effect size in the one-vs.-all comparison were also considered for interpretation of the distinguishing profile for each experience level class. All data analyses and variable calculations were performed using a custom-written MATLAB script (MathWorks, Natick, MA, United States).

For a better understanding of the discrete GRF variables that were included as possible inputs to the model they were compared between the experience groups using ANOVAs followed by Bonferroni *post hoc* tests (*P* < 0.05). The significance level was set at 5%. All the univariate analyses were performed with SPSS Statistics 23.0 (IBM, Armonk, NY, United States).

## Results

### Performance of SVM Classification Models

All classification performance measures are presented in Table 2. The C-parameter values influenced the SVM performance, and the best model for classifying the less experienced group used C = 100, while the best classifiers for the other groups used C = 1. The SVM models for the classification of all experience groups presented a cross-validated accuracy that surpassed the minimum significant accuracy indicated by the critical binomial test. The best classification model for the less experienced group obtained the highest MCC value and the best balance between precision and recall rates, rendering an F1-score of 0.80. The best model for the moderately experienced presented a perfect precision rate, wherein all the runners identified as a member of this group were correctly classified, but almost half of the moderately experienced runners were misclassified, resulting in low recall rate (56.5%). The model for the experienced group presented a lower accuracy rate, but precision and recall rates reached 82.1 and 76.7%, respectively. Overall, the best classification models achieved an MCC score of strong to very strong relationships.

**TABLE 2:** Performance measures of the SVM models.

	Less experienced vs. all	Moderately experienced vs. all	Experienced vs. all
**Minimum significant accuracy Cross-validation performance**	76.9%	78.2%	70.5%
*Accuracy*	88.5%	87.2%	84.6%
*Recall*	72%	56.5%	76.7%
*Precision*	90%	100%	82.1%
*F1-score*	0.80	0.72	0.79
*MCC*^a^	0.73	0.69	0.67

*^*a*^Matthew’s correlation coefficient.*

### Discriminating Running Patterns

**FIGURE 2:**
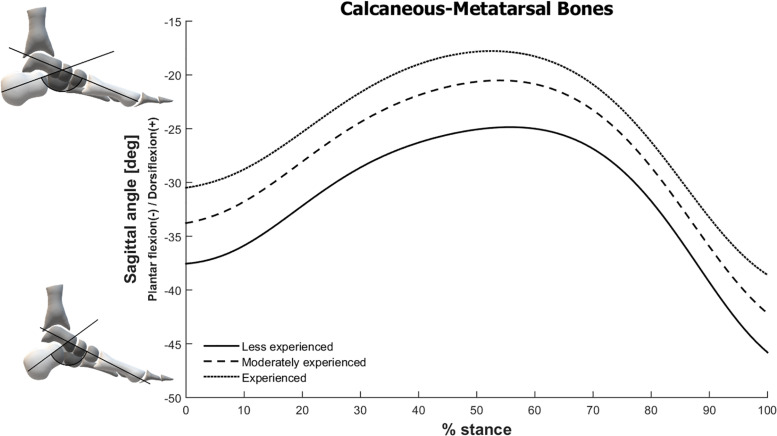
Angle between calcaneus and metatarsal bones in the sagittal plane during stance phase of running for all three running experience level groups. The foot schematics in the left represent the calculated angles.

**FIGURE 3:**
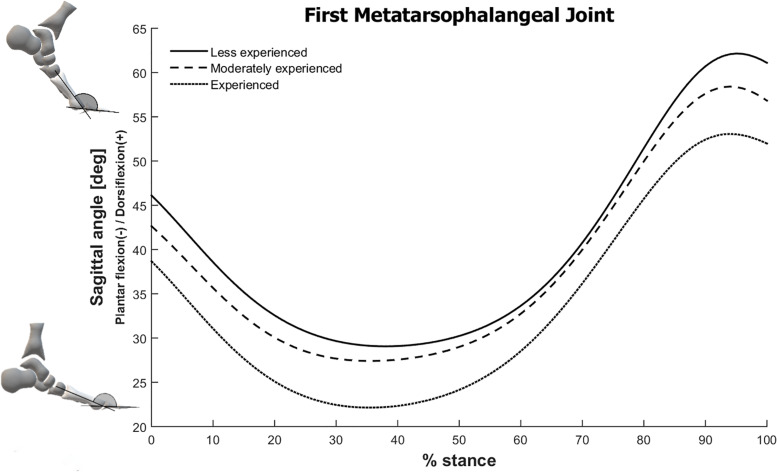
First metatarsophalangeal joint sagittal plane angle during stance phase of running for all three running experience level groups. The foot schematics in the left represent the calculated angles.

The SVM model for the less experienced group selected 81 variables as the input features, from which 72 of them were PCs from the foot-ankle kinematic and kinetic (ankle sagittal moment and power) time series, with a total variance explained of 99.9% The other nine features were GRF variables, which included the loading rate, 1^st^ and 2^nd^ vertical peaks, both vertical impulses, the anteroposterior negative and positive peaks, and impulses from decelerating and accelerating phases. However, between-group comparisons of the GRF variables did not find any significant differences across experience levels (Table 3). The reconstructed waveforms indicated that the kinematic features considered important for the identification of the less experienced runners were mainly related to greater Cal-Met plantar flexion, and greater Met-Hal dorsiflexion (Figures 2, 3). Both of these joint movements are related to PC2, which was the first feature included in the forward feature selection process and represents 15.5% of variance explained of the total foot-ankle data, wherein the relative proportion of representation is highly loaded on Cal-Met (44.1% of PC2) and Met-Hal (28.1%) sagittal planes.

**TABLE 3:** Mean and standard deviation of ground reaction force variables extracted from the stance phase of running and results from the between-group comparisons and correlation analysis.

Discrete variable	Less experienced (*n* = 24)	Moderately experienced (*n* = 23)	Experienced (*n* = 31)	ANOVA *F*	*P*
**Vertical component**					
Loading rate (N/s)	78.86 ± 43.45	61.94 ± 25.95	69.00 ± 36.79	1.295	0.28
First peak (BW)	1.19 ± 0.40	1.09 ± 0.37	1.08 ± 0.39	0.598	0.55
Second peak (BW)	2.15 ± 0.29	2.26 ± 0.30	2.21 ± 0.28	0.990	0.38
Impulse 1 (N.s)	0.033 ± 0.020	0.027 ± 0.016	0.031 ± 0.019	0.590	0.56
Impulse 2 (N.s)	0.318 ± 0.028	0.327 ± 0.024	0.311 ± 0.030	1.898	0.16
**Anteroposterior component**					
Positive peak (BW)	0.226 ± 0.050	0.238 ± 0.045	0.239 ± 0.053	0.502	0.61
Negative peak (BW)	−0.230 ± 0.080	−0.226 ± 0.614	−0.236 ± 0.053	0.160	0.85
Impulse 3 (N.s)	−0.017 ± 0.006	−0.018 ± 0.005	−0.016 ± 0.005	1.027	0.36
Impulse 4 (N.s)	0.016 ± 0.004	0.016 ± 0.004	0.017 ± 0.005	0.254	0.78

*BW, body weight.*

The classification model for the moderately experienced group selected eight PCs that were responsible for only 0.4% of variance explained, and two GRF variables, which were the loading rate and anteroposterior negative peak. The foot-ankle mechanics features distinguishing the moderately experienced runners were not as evident, since they were only PCs representing very low variance explained and did not present a visually identifiable pattern.

For the experienced group the best model selected six foot-ankle kinematic/kinetic waveform PCs representing 21.7% of variance explained, along with the 1^st^ vertical GRF peak and the anteroposterior impulse from the acceleration phase. The experienced runners presented the opposite distinguishing features from the less experienced group, which were mainly related to smaller CalMet plantar flexion and smaller Met-Hal dorsiflexion (Figures 2, 3). These differences were also related to PC2, which was the only selected with a relatively high variance explained.

## Discussion

The main purpose of the study was to determine if running experience level could be classified based on foot-ankle kinematic and kinetic patterns, and GRF variables. The results of this study showed that this classification is possible since the SVM models successfully separated all experience groups, with the less experienced and experienced runners presenting discriminating features with opposing motor patterns in the metatarsophalangeal and calcaneus-metatarsal joints, while the moderately experienced group did not present an explicitly visible pattern, although they were classified with significant accuracy.

One of the distinguishing kinematic features for the less experienced runners was a larger toe dorsiflexion angle throughout the whole stance phase. It is possible that the lack of experience is related to a greater use of hallux and toe extensor muscles in combination with tibialis anterior muscle as ankle dorsiflexors, in order to guarantee toe clearance and dorsiflexion throughout the running stance phase, especially during the weight acceptance phase, which could result in the observed greater toe dorsiflexion. Interestingly, the experienced group presented smaller toe dorsiflexion angles than the other runners, suggesting that there is a change in motor strategy with skill acquisition, possibly reducing muscle activation that are unnecessary and causing less energy consumption. There is evidence that runners with more years of running experience show different lower-limb coordination patterns, more specifically, in the variability of coordination, as measured by non-linear analysis (Agresta et al., 2019; Hafer et al., 2019; Mo and Chow, 2019), showing that deliberate practice seems to cause motor strategy adaptations that modifies biomechanical patterns. Unfortunately, to our knowledge there are no studies comparing muscular activation patterns in runners with different experience levels showing evidences of that. A study in cyclists showed that novice cyclists present longer periods of primary activity in leg muscles and more extensive coactivation between muscles, as opposed to trained cyclists, who display shorter bursts with consistent timing of muscle activity (Chapman et al., 2008). Although cycling is a different motor task, this finding suggests that deliberate practice of a motor task seems to change muscle coordination.

The metatarsophalangeal joints function as a dissipater of large amounts of energy during running and sprinting, particularly when a passive dorsiflexion occurs at the foot contact transition from the metatarsal heads onto the toes, but fail to generate any energy at push off by remaining in that position (Stefanyshyn and Nigg, 1997). Since the less experienced runners displayed larger angles of hallux dorsiflexion during this transition between midstance to push off, they would be expected to have greater soft tissue tension, such as in the toe flexor muscles and tendons, and plantar fascia (Bruening et al., 2018). However, since there was little or no metatarsophalangeal plantar flexion, there was more energy dissipated by the passive structures, leading to less efficient propulsion in the following phase.

There was also an opposing behavior between the less experienced and experienced groups regarding the calcaneus-metatarsal, with the former displaying greater plantar flexion. This higher plantar flexion in less experienced runners could be associated to the greater toe dorsiflexion angles, because of the windlass mechanism, in which the dorsiflexion moments at the metatarsophalangeal joints cause tension to the plantar aponeurosis, pulling the calcaneus toward the metatarsal heads (Holowka and Lieberman, 2018).

Evidence shows that the passive structures of the foot arch have an important role in the metabolic energy-saving of the foot by the reduction of the mechanical work that would be needed from muscle activation (Stearne et al., 2016). One of the exclusion criteria for the current study was the use of minimalist shoes, which are known to be associated with higher intrinsic foot muscle volume after a running training regime (Miller et al., 2014; Chen et al., 2016). Since the subjects of this study all ran with traditional shoes, the function of the foot intrinsic muscles is probably reduced due to the support given to the medial longitudinal arch and the stiffness of the midsole in the traditional running footwear, possibly causing a higher reliance on the passive properties of the plantar tissues. Therefore, it is expected that there would be no disparity in the function of these muscles between groups. The fact that the experienced runners presented a less plantar flexed calcaneus-metatarsal joint potentially indicate the presence of a higher tension in the plantar structures, such as the plantar fascia, and intrinsic and extrinsic foot muscles, since this higher angle would correspond to a greater distance between the rearfoot and the forefoot. Although the arch compression/recoil property is not changed due to experience level, since the calcaneus-metatarsal range of motion is similar between all experience groups (Figure 2), the foot plantar structures would be working in a position with higher strain in the experienced runners. If experienced runners rely more on the passive structures, less muscle activation is needed, and less energy is spent (Stearne et al., 2016), showing that experienced runners might have a more efficient foot-ankle biomechanical pattern. The question remains whether this pattern could be related to protective factors against injuries in runners with more experience.

Moderately experienced runners did not show a clearly distinct biomechanical pattern since the SVM model selected only higher order PCs with low explained variance (0.4%) as discriminating factors, indicating that differences were very subtle and complicated to be visualized. Still, these complex patterns were able to successfully distinguish this particular group from the other runners. It is possible that this is a transitioning group that is composed of a more heterogeneous sample, which would hinder the identification of a specific movement pattern. This model presented a perfect precision rate, i.e., all the subjects identified as moderately experienced runners were correctly classified. However, it had a low recall rate (56.5%), meaning that it fails to detect almost half of the runners from the moderately experienced group. Therefore, there is a specific mechanical pattern for this group, which is probably responsible for the high precision rate of the model, but the high rate of false negatives supports the assumption of a heterogeneous and intermediate group, with a great portion of runners possibly behaving similarly to either experienced or less experienced runners. It is possible that a further stratification of the experience levels could improve the discrimination among these subjects, but that would not be possible with the current sample size, which is a limitation of this analysis.

Although there were no significant differences between experience levels for the GRF variables, they were still crucial for the performance of the classification models, since they were selected as discriminating features by the SVM models. This shows that the combination of different biomechanical features is a better representation of the motor behavior and necessary for an improved identification of the mechanical patterns. Unfortunately, it was not possible to identify how these GRF factors are specifically related to the distinguishing mechanical pattern within each group.

The identified mechanical patterns should be considered with caution, since the participants were asked to run barefoot because it was needed for the multi-segment foot model implementation (Leardini et al., 2007; Portinaro et al., 2014), which could have altered the runners’ habitual movement. Nevertheless, they still reflect distinct motor strategies across experience levels, highlighting the importance of foot-ankle mechanics to discriminate the experimental groups. On a similar matter, we did not define a fixed running velocity for the subjects, which could lead to mechanical differences that are due to speed effects. However, there were no significant differences in running velocity across experience levels, thus we do not expect that such effects influenced our results.

A PCA was applied to the foot-ankle kinematic and kinetic data for dimensionality reduction and feature extraction purposes, and this strategy allows the identification of patterns across the foot joints. However, all the PCs were included as possible features in the SVM, even though the movement patterns are only visible in the low-order PCs and the high-order PCs could be including noise and signal artifacts. This approach was chosen because there are studies that were not able to identify differences between experience levels, indicating that the changes due to running experience could be more complex and subtle, and such differences have been shown to be identified only by the high-order PCs (Phinyomark et al., 2015). Although it is possible that a portion of the PCs are representing noise in the data, the SVM models still successfully classified the different experience levels by including these high-order PCs, and presented an accuracy rate that surpassed the minimal threshold determined by a critical binomial test.

Another limitation of this study is its relatively small sample size, since the training data set for a machine learning classifier approach should increase exponentially in size for each added input dimension (Altman and Krzywinski, 2018). For that reason, the SVM was chosen for the analysis, because it considers the data points in the margins of each class to determine the separating hyperplane, thus it is not influenced by the distribution of the data points and can effectively separate binary classes even with limited sample size (Noble, 2006). In addition, the use of SVM has shown very high classification performance in clinical settings (Golub et al., 1999; Son et al., 2010) and with biomechanical data (Lai et al., 2009; Fukuchi et al., 2011).

Another problem that a reduced sample size can encounter is data overfitting, which causes the classifying function to be too specific to the training dataset and not generalizable. Although it would be ideal to have a test dataset to ensure the generalizability of the analysis, it was not possible to use this strategy because of the limited sample size coming from the major randomized clinical trial. Thus, in order to avoid overfitting, a 10-fold cross-validation was applied in the feature selection process and the performance measurement, increasing the robustness of the results, and a linear kernel was applied to the algorithm, again to prevent overfitting by not adapting the hyperplanes to irregular margins. Still, the current results should be considered with caution since extrapolation for general population may not be suitable. Furthermore, since SVM is intended for binary classifications, it was necessary to use a one-vs.-all approach, which causes the group sizes to be unequal and interferes in the classifier performance measures. Nevertheless, this issue was dealt by applying the MCC as the main performance measure because it is more reliable and informative when evaluating binary classifications, especially on imbalanced datasets (Chicco and Jurman, 2020).

This is the first study to apply a machine learning approach to investigate how running experience affects foot-ankle biomechanics. The results indicate that using foot-ankle kinematic and kinetic waveforms associated with GRF variables as inputs in an SVM classifier can successfully separate and classify runners with different levels of experience. The main identifiable features that are important for the discrimination were the toe dorsiflexion and calcaneus-metatarsal plantar flexion angles. The less experienced group presented greater metatarsophalangeal dorsiflexion throughout the whole stance phase, which could cause higher energy dissipation and a less efficient propulsion. The more experienced group displayed smaller calcaneus-metatarsal plantar flexion that might be related to a more efficient biomechanical pattern regarding energy expenditure. As for the moderately experienced runners, although they were successfully classified, they did not present a visually identifiable running pattern, and seem to be an intermediate group between the less and more experienced runners. The current findings can potentially guide the development of training programs and rehabilitation protocols aimed at foot-ankle mechanics for different levels of experience in running. Furthermore, future studies could investigate what are the implications of those different biomechanical patterns according to the experience level in running performance and injuries.

## Data Availability Statement

The raw data supporting the conclusions of this article will be made available by the authors, without undue reservation, to any qualified researcher.

## Ethics Statement

The studies involving human participants were reviewed and approved by the Research and Ethics Committee of the School of Medicine of the University of São Paulo (Protocol no. 031/15). The participants provided their written informed consent to participate in this larger randomized clinical trial registered at ClinicalTrials.gov (Identifier NCT02306148).

## Author Contributions

All authors have made substantial contributions to the manuscript. ES, RW, and IS were responsible for the conception and design of the study. AM was responsible for data acquisition and data processing. ES and RW were responsible for data analysis and interpretation, and drafting the article. IS and AM revised the manuscript critically. All authors read, provided feedback, and approved the submitted version.

## Conflict of Interest

The authors declare that the research was conducted in the absence of any commercial or financial relationships that could be construed as a potential conflict of interest.
